# The Effects of Network Neighbours on Protein Evolution

**DOI:** 10.1371/journal.pone.0018288

**Published:** 2011-04-12

**Authors:** Guang-Zhong Wang, Martin J. Lercher

**Affiliations:** 1 Institute for Computer Science, Heinrich-Heine-University, Düsseldorf, Germany; Centre for Genomic Regulation (CRG), Universitat Pompeu Fabra, Spain

## Abstract

Interacting proteins may often experience similar selection pressures. Thus, we may expect that neighbouring proteins in biological interaction networks evolve at similar rates. This has been previously shown for protein-protein interaction networks. Similarly, we find correlated rates of evolution of neighbours in networks based on co-expression, metabolism, and synthetic lethal genetic interactions. While the correlations are statistically significant, their magnitude is small, with network effects explaining only between 2% and 7% of the variation. The strongest known predictor of the rate of protein evolution remains expression level. We confirmed the previous observation that similar expression levels of neighbours indeed explain their similar evolution rates in protein-protein networks, and showed that the same is true for metabolic networks. In co-expression and synthetic lethal genetic interaction networks, however, neighbouring genes still show somewhat similar evolutionary rates even after simultaneously controlling for expression level, gene essentiality and gene length. Thus, similar expression levels and related functions (as inferred from co-expression and synthetic lethal interactions) seem to explain correlated evolutionary rates of network neighbours across all currently available types of biological networks.

## Introduction

Recently, there has been increased interest in the influence of biological networks on protein evolution. Network connectivity, i.e., the number of connections that an individual protein has, was the first parameter reported to influence protein evolution [1], [2], [3], [4], [5]. A negative correlation between connectivity and evolutionary rate was observed not only in protein-protein interaction networks [1], , but also in metabolic [5], co-expression [6], and genetic interaction networks [7]: genes with more interaction partners appear to evolve more slowly. However, in particular in the case of protein interaction networks, these effects are rather weak [3], [8], [9], [10]. Furthermore, apparent network effects may be artefacts caused by biases in the available datasets [11], [12], [13], or by co-variation of network properties with other variables [10], [14].

In protein-protein interaction networks, another network parameter, betweenness was found to be correlated with evolutionary rate: proteins with high betweenness (more ‘central’ proteins) tend to evolve more slowly [15]. A corresponding effect of centrality was also seen in the metabolic network of yeast [5]. In contrast, transcription factors that are more central in the regulatory network evolve faster than other genes [16], confirming that transcription networks have differ drastically from other biological networks. Again, the effect in protein interaction networks has been attributed to co-variation of network properties with other variables, in particular with gene expression level [14], [17].

Thus, evidence for a direct influence of network structure on the rate of sequence evolution is controversial and appears rather weak. Are there other features in the network that influence evolutionary rates? Here, we study the relationship between the evolutionary rate of a given protein and the evolutionary rate of its network neighbours. It has been reported that in the protein-protein interaction network, interacting proteins tend to have similar evolutionary rates [1], [18], [19], [20], [21], [22]. There is an ongoing debate if this correlated evolution of physically interacting proteins is caused by compensatory mutations between binding partners (co-evolution), or if it is simply due to similar selective constraints, like those resulting from similar expression levels. Careful studies of small sets of proteins have confirmed that co-evolution of interacting binding sites does indeed occur [18], [21], [23]. An investigation of the three-dimensional structures of about 100 yeast proteins indicated that buried residues – which are located on a stable interaction surface between protein units – are under stronger evolutionary constraints than solvent exposed sites [24], even after excluding the effect of expression level. Moreover, residues close to the binding sites responsible for protein-protein interactions show higher co-evolution signals than residues outside the binding region [25]. However, another analysis observed that correlations purely based on the co-evolution of proteins surfaces and binding interfaces are not higher than the correlation when considering the complete sequences of interacting proteins [22]. One potential mechanism promoting similar evolutionary rates of physically binding proteins could be similar fractions of residues involved in protein-protein binding. These residues show reduced evolutionary rates, both due to their decreased solvent accessibility, and due to the involvement in binding *per se* [26]. However, the directly interacting residues constitute only about 10% of the total sequence [21], and not all of these contribute strongly to the binding energy. Thus, correlated evolution measured at the whole-sequence level is probably not explained by direct co-evolution at the binding interfaces [22], [27].

Is correlated evolution of network neighbours also found in other types of biological networks? If the protein and its network partners co-evolve or co-adapt [28], we indeed expect that the partners show similar rates of evolution. For example, in the protein-protein interaction network, interacting binding sites usually show co-evolution [18], [21], [23], [25]. Physically interacting human proteins (*i.e.*, neighbours in the protein-protein interaction network) show stronger signs of correlated evolution than proteins in the same biochemical pathway (*i.e.*, neighbours in the metabolic network) [29]. In co-expression networks, neighbouring genes are often involved in the same biological function, and in genetic interaction networks, the mutation of one protein changes the fitness effects of mutations in its partners; thus, it appears likely that neighbours in these networks also co-evolve. By comparing the number of substitutions per site between interacting proteins, we tested the strength of correlated evolution in the yeast protein-protein interaction, co-expression, metabolic, genetic interaction, and transcriptional regulatory networks.

From an analysis of the evolution rate of each focal protein in the network and the mean rate of its neighbours, we show that there is indeed a positive – although weak – neighbour correlation in evolutionary rate for most biological networks. Further, we find that the correlation can be mostly explained by shared evolutionary constraints, in particular related to similar expression levels. These results support the view that the co-evolution of binding sites or functional similarity plays only a minor role in determining network effects on overall protein evolution. Interestingly, we find that co-expression implies correlated evolution independently of other known predictors of evolutionary rate.

## Results

### Proteins evolve at similar rates as their network neighbours

A number of independent studies have confirmed that physically interacting proteins evolve at similar rates. We first make sure that we can recover this observation using an updated protein interaction data set and our modified methodology. In order to ensure that all protein-protein interactions in the dataset refer to direct contact between proteins, protein interactions within the same complex but without direct contact were excluded.

**Figure 1:**
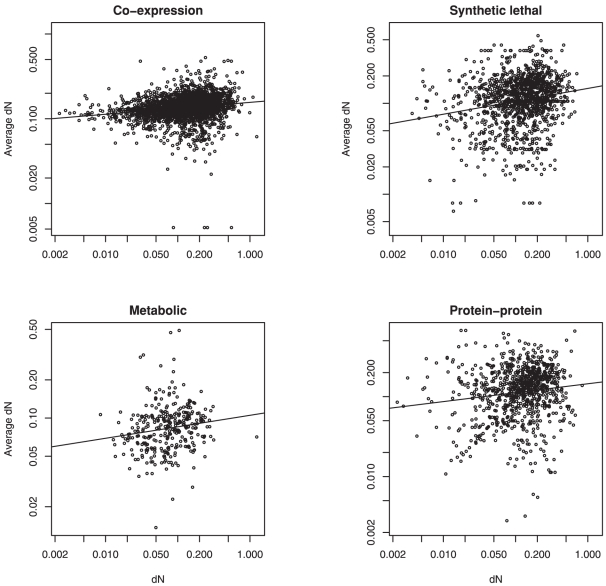
Correlations between the evolutionary rate *dN* of focal proteins and the average rate of their network neighbours neighbours for four different types of interaction networks.

We considered each protein in turn as the ‘focal’ protein, and calculated the average evolutionary rate across its direct network neighbours. If adjacent proteins show similar evolutionary rates, we would expect a positive correlation between the evolutionary rate of the focal protein and the average neighbour rate. We indeed found the expected correlation in the protein-protein interaction data (Figure 1; for *dN*, Spearman's rank correlation coefficient *ρ* = 0.15, *p* = 3.7×10^−6^; for *dN*/*dS*, *ρ* = 0.14, *p* = 2.1×10^−5^).

We thus confirmed that neighbouring proteins in the yeast protein-protein interaction network evolve at similar rates. Is this correlation a general feature of all biological networks? If all types of interactions impose constraints on sequence evolution, this correlation would generally be expected. To test this hypothesis, we used recently published yeast network data, encompassing co-expression data [30], genetic interaction data [31], transcription regulation data [32], and metabolic data [33]. After removal of duplicated links, we obtained final datasets with 14,283 interactions in the metabolic network, 12,873 interactions in the transcription network, 13,030 interactions in the synthetic lethal interaction network, and 689,100 interactions in the co-expression network. Note that for our first analysis of genetic interactions, we only chose synthetic lethal interactions; below, we also analyze a much larger data set of non-lethal genetic interactions.

As seen in Table 1, except for the transcription regulation network, each of the biological networks exhibits a significant correlation between the evolutionary rates of focal proteins and the average evolutionary rates of their neighbours (*p*<0.002 from comparison to random pairs in each case). These correlations are still relatively weak (Spearman's *ρ* between 0.18 and 0.27 for *dN*), but are somewhat stronger than those seen for the protein-protein interaction network. Thus, interacting neighbours show statistically significant similarity in their evolutionary rates for all available genome-scale networks in yeast, with the sole exception of the regulatory network.

**Table 1:** Significant correlations between the evolutionary rates of proteins and the average rates of their network neighbours, except for the transcription regulation network.

	*dN* vs. neighbour *dN*		*dN*/dS vs. neighbour *dN*/dS		*dN* vs. connectivity	
Interaction type	*ρ* 1	*p*	*ρ*	*p*	*ρ*	*p*
Protein-protein	0.15	3.7×10^−6^	0.14	2.1×10^−5^	−0.059	0.047
Synthetic lethal	0.18	6.2×10^−11^	0.16	8.5×10^−9^	−0.058	0.021
Metabolic	0.21	1.6×10^−4^	0.18	0.0017	−0.18	0.0014
Co-expression	0.27	<10^−15^	0.23	<10^−15^	−0.0055	0.80
Regulation	−0.02	0.34	−0.02	0.50	−0.28	<10^−15^

1 Spearman's rank correlation coefficient.

For the transcription regulation network, there is no significant neighbour correlation in evolutionary rates (Table 1). This may be rooted in a fundamental difference between the regulatory network and the other network types considered here: connections in the transcriptional network are strongly asymmetrical. Our results indicate that the sequence evolution of transcription factors is decoupled from their target genes. This lack of correlation may partly stem from the fact that network rewiring is the main evolutionary force of transcription regulation [34].

In addition to the synthetic lethal genetic interaction data, which is based on literature surveys, we also analysed a more recent genetic interaction dataset from a large high-throughput experiment [7]. Only interactions fulfilling a stringent cut-off criterion were used in order to ensure high data quality. In contrast to the findings reported in Table 1 for the synthetic lethal interactions, we did not observe any significant correlations between the evolutionary rates of network neighbours, neither for the total network (including both positive and negative interactions), nor for negative interactions alone (total network: *p* = 0.30, *ρ* = 0.024; negative interactions: *p* = 0.31, *ρ* = 0.024). Thus, it may be that only synthetic lethal interactions have an influence on protein evolution, while weaker (or positive) interactions do not.

### The influence of network neighbourhoods on evolution is largely explained by expression level

While our preliminary analysis shows that in most of the networks, neighbouring genes have similar evolution rates, these correlations may not be causal, but may stem from the influence of other correlated (confounding) variables. Indeed, in the protein-protein interaction network, Agrafioti *et al.* found that most of the correlation can be attributed to similarities of the neighbours in expression level [10], with additional contributions from correlated functions and involvement in biological processes as inferred from GO annotations. Another parameter one might think of in this context is network connectivity (the number of direct neighbours) [10], as some previous analyses found that connectivity influences evolutionary rates in various networks. For the different network types analysed here, we confirmed a weak but significant negative correlation between connectivity and evolutionary rate *dN*, with the transcriptional regulation network again being the only exception (Table 1).

However, these weak correlations with connectivity are not sufficient to explain the observed correlations among network neighbours. After controlling for connectivity using partial regression analysis, only the correlation between neighbours in the metabolic network became non-significant (Table 2). Thus, connectivity cannot generally explain why neighbouring proteins evolve at correlated rates.

**Table 2:** Correlation between *dN* and average *dN* of the neighbours after controlling separately for protein abundance, codon usage (CAI), or mRNA expression level; and after simultaneously controlling for all three expression measures and for protein length, gene essentiality, and network connectivity using a linear model.

	Controlling for:									
	Protein abundance		Codon usage		mRNA expression		Connectivity		6 variables in combined linear model	
Interaction type	*r* 1	*p*	*r*	*p*	*r*	*p*	*r*	*p*	% explained^2^	*p*
Protein-protein	0.068	0.083	0.031	0.41	0.059	0.08	0.074	0.025	-	-
Synthetic lethal	0.13	5×10^−5^	0.10	0.0003	0.14	6×10^−7^	0.14	4×10^−7^	1.3 (0.4–2.9)	0.00094
Metabolic	0.014	0.81	−0.040	0.53	0.0034	1.0	0.028	0.62	-	-
Regulation	−0.013	0.70	−0.023	0.46	−0.017	0.52	−0.005	0.84	-	-
Co-expression	0.20	<10^−15^	0.143	3×10^−15^	0.17	<10^−15^	0.19	<10^−15^	2.2 (1.3–3.5)	4.4×10^−6^

1 Partial regression coefficient.

2 Percent of variation in *dN* explained by average neighbour *dN* independently of the other variables, and 95% confidence intervals (calculated using a relative importance measure that averages over orderings of regressors, with confidence intervals based on 1000 bootstraps [43]). This combined analysis was only performed if controlling for individual variables did not remove the correlation with *dN*.

The most important factor determining yeast protein evolutionary rates is gene expression level [35]. Principal component regression analysis has shown that expression-related variables explain nearly half of the variation in protein evolutionary rate among yeast proteins [8]. Thus, two interacting proteins might show signs of correlated evolution just because they have similar expression levels. Indeed, two previous analyses found that correlated evolution of network neighbours is not due to compensatory mutations between binding interfaces, but that similar expression levels account for most of the co-evolution [10], [22]. Do similar expression levels of interacting genes more generally explain the co-evolution of neighbours in biological networks?

It is widely accepted that there are three variables that measure aspects of gene expression in yeast: mRNA expression level, codon usage bias (measured, *e.g.*, as codon adaptation index, CAI), and protein abundance [8]. After controlling for expression level using any one of these three factors, both the protein-protein interaction network and the metabolic network do not show any significant correlations among neighbours anymore.

In contrast, both the synthetic lethal interaction and the co-expression network still exhibit highly significant correlations between neighbours' evolutionary rates even after controlling for similar absolute expression levels (Table 2). While it may seem confusing that we control co-expression for expression level, note that co-expression is defined as correlated up- and down-regulation across measurements in time-course experiments. Thus, two genes *A* and *B* would be perfectly co-expressed if the number of transcripts of *A* was always a fixed multiple of those of *B*. This means that high co-expression does not necessarily imply similar absolute expression levels. A statistically significant evolutionary rate correlation between co-expressed and genes remains even after we additionally control for two further potential confounding factors, protein length and gene essentiality, even if co-expression explains only about 2% of the variation in evolutionary rate; a similar result is seen for genes with synthetic lethal interactions (Table 2).

Thus, all network effects on protein evolution appear to be mediated by gene expression – either directly through co-expression, or indirectly through similar expression levels of interacting partners – or by strong negative genetic interactions. This effect may not be unique to yeast: recently, it was shown that co-expression also influences protein evolution rate in humans [36].

## Discussion

Neighbouring proteins in yeast interaction networks – with the exception of the strongly asymmetric transcriptional regulation network – evolve at correlated rates. While the observed correlations are statistically significant, their magnitude is generally small: even when not controlling for expression level and other confounding variables, network neighbourhood explains only between about 2% and 7% of the variation in the non-synonymous substitution rate *dN* (Table 2). By controlling for other factors that constrain protein evolution, others have previously shown that similar expression levels are sufficient to explain most of the correlated evolutionary rates in the protein-protein network [10], [22]. We found that the same is true in the metabolic network, but not in the co-expression and synthetic lethal genetic interaction network. Thus, strong negative genetic interactions appear to be more informative about evolutionarily relevant functional similarity than protein-protein interactions or neighbourhood in the metabolic network. Further, it appears that neighbouring genes in different types of networks evolve at somewhat similar rates largely because they have similar absolute expression levels or because they are co-expressed.

Genes with a synthetic lethal interaction can compensate for each others loss, suggesting that they can perform (at least partially) identical biological functions. Similarly, co-expressed genes often have correlated functions. Thus, our results suggest that the weak signs of correlated evolution are not a mysterious emergent property of networks, but rather a consequence of similar absolute expression levels and of correlated function. In this sense, our results generalize previous observation on the yeast protein-protein interaction network [10] to other types of biological networks.

## Methods

### Evolutionary rates

The evolutionary rates of yeast genes (*dN*, the number of non-synonymous substitutions per non-synonymous site, and *dN*/*dS*, *dN* divided by the number of synonymous substitutions per synonymous site) were obtained from a comparison of 4 closely related yeast species including *Saccharomyces cerevisiae* [37]. In the main text, we refer to *dN* to represent the evolutionary rate of yeast protein coding sequences. Alternatively using *dN*/dS does not change the results.

### Network data

All network and other data is for the yeast *Saccharomyces cerevisiae*. For all networks, only genes for which evolutionary rate values are available were considered.

The co-expression network was obtained from a combination of 40 time-series microarray experiments [30]. Pearson's correlation coefficient *r* across all experiments was used as a measure of the co-expression level of two genes. Two genes are linked in the resulting co-expression network if their expression profiles are correlated with *r*> = 0.5. Note that co-expression reflects correlated relative changes in expression level across time points; it does not necessarily imply similar absolute expression levels.

Protein-protein interaction data was obtained from the CCSB interactome database (http://interactome.dfci.harvard.edu/index.php?page=home). To ensure high data quality, literature-based interactions (LC-multiple), as well as co-complex associations for which we are not sure if the two proteins are in direct contact with each other (Combined-AP/MS), were excluded. In total, we obtained four datasets (CCSB-YI1, Ito-Core, Uetz-Screen and Y2H-Union), containing a total of 6,273 protein-protein interactions. We built the union of these four sets, removing duplicate interactions. This led to 4,349 interactions in the final data set.

A synthetic lethality (strong negative genetic interaction) network was extracted from BIOGRID, version 2.0.60 [31]. Only interactions tagged with “Synthetic Lethality” were used, resulting in a total of 15,196 interactions. After removing duplicate interactions, we obtained a final data set of 13,030 interactions. Another genetic interaction data set was published recently [38]. From this, only interactions below a stringent cutoff [38] were used, resulting in a second set of 74,984 interactions.

The yeast metabolic network was obtained from Ref. [33] and compiled according to the procedure previously reported [5]. After removing duplicate interactions, we retained 11,179 interactions in our dataset (14,283 in the raw data).

### Other datasets

Protein abundance in log-phase growth were taken from Ref. [39], yeast mRNA expression levels from Ref. [40], and codon adaptation index (CAI) from Ref. [37]. Protein length was calculated based on the protein sequences given in SGC [41]. The identity of more than 1,100 essential genes was obtained from the Saccharomyces Genome Deletion Project web page (http://yeastdeletion.stanford.edu/).

### Statistical analyses

All statistical analyses were performed using the statistical software environment *R* [42]. Partial regression analysis was performed using an R script from Ref [8] as described therein. For Table 2, percent of variation explained was calculated using a relative importance measure that averages over orderings of regressors, with confidence intervals based on 1000 bootstraps [43].
